# Image Based Hair Segmentation Algorithm for the Application of Automatic Facial Caricature Synthesis

**DOI:** 10.1155/2014/748634

**Published:** 2014-01-29

**Authors:** Yehu Shen, Zhenyun Peng, Yaohui Zhang

**Affiliations:** ^1^Department of System Integration & IC Design, Suzhou Institute of Nanotech & Nanobionics, Chinese Academy of Sciences, Suzhou 215125, China; ^2^Suzhou Wowman Information Services CO., LTD., Suzhou 215123, China

## Abstract

Hair is a salient feature in human face region and are one of the important cues for face analysis. Accurate detection and presentation of hair region is one of the key components for automatic synthesis of human facial caricature. In this paper, an automatic hair detection algorithm for the application of automatic synthesis of facial caricature based on a single image is proposed. Firstly, hair regions in training images are labeled manually and then the hair position prior distributions and hair color likelihood distribution function are estimated from these labels efficiently. Secondly, the energy function of the test image is constructed according to the estimated prior distributions of hair location and hair color likelihood. This energy function is further optimized according to graph cuts technique and initial hair region is obtained. Finally, K-means algorithm and image postprocessing techniques are applied to the initial hair region so that the final hair region can be segmented precisely. Experimental results show that the average processing time for each image is about 280 ms and the average hair region detection accuracy is above 90%. The proposed algorithm is applied to a facial caricature synthesis system. Experiments proved that with our proposed hair segmentation algorithm the facial caricatures are vivid and satisfying.

## 1. Introduction

Hair region is one of the most important components in human face. It is one of the main reasons for the different appearances among different people. Reference [1] argued that hair region provides cues during the face recognition process for humans. Reference [2] proved that hair region is the most important feature during the recognition of familiar people according to experiments. In spite of face recognition, hair segmentation is also the key component in automatic facial caricature synthesis systems. It is pointed out in [3] that hair has great impact on face animation.

Although hair features contain several aforementioned benefits, most of the current face recognition algorithms only use features in face region without considering the hair [4]. For the application of facial caricature synthesis, hair region is usually extracted manually or automatically detected with a bright and homogeneous background. This is because the shape of hair varies widely and the color is also different among different people. Furthermore, complex lighting conditions and backgrounds complicate the process of hair segmentation.

For the above-mentioned reasons, there are only a few researches about image based hair segmentation techniques. Yacoob and Davis [5] are pioneers who made systematic researches about hair detection. They construct a simple hair color model and detect hair region through region growing technique. Yacoob and Davis aim at the analysis of hair length, volume, color, symmetry, and the effect of hair region in the face recognition process. As a result, hair detection algorithm proposed in [5] is quite simple and only applicable to images with homogeneous backgrounds. Lipowezky et al. [6] improve the method proposed in [5]. They fuse color and texture information and represent hair region through fuzzy mask for the application of virtual hair recolorization. However, this paper only provides qualitative results and lacks quantitative analysis. The computational cost which is about 5 minutes per image is also quite high since complex image matting technique is applied in this algorithm. Rousset and Coulon [7] estimate the initial hair region binary mask through color information and frequency domain analysis. Then they also use image matting technique in order to get the final hair region mask. The average processing time for an image of 200 × 300 is about 20 seconds on a 3 GHz CPU. For the application of facial caricature synthesis and face animation, Chen et al. [8] use manually labeled hair line together with knockout image matting technique for hair detection. Wan et al. [9] propose to combine image thresholding and contour tracking techniques to segment hair regions. This method is very simple but could hardly deal with complex backgrounds. Recently, Wang et al. [10] propose a compositional exemplar-based model for generating probabilistic hair mask in the manner of divide-and-conquer. In [11], a coarse-to-fine hair segmentation method is proposed. They firstly combine active segmentation with fixation and mean shift technique to get hair seeds. Then an SVM classifier is learnt online in order to predict the hair likelihood probability. It could deal with heads in different poses. Although the above two methods showed promised results, their methods are very complex and hard to implement. Furthermore, both methods need sophisticated training procedure which is very time consuming. For example, it is reported in [10] that the training time is 95 hours without considering human labeling.

Despite the above-mentioned automatic hair segmentation algorithms, high quality semiautomatic segmentation methods gradually emerge recently. Li et al. [12] proposed a famous interactive segmentation tool which usually needs a few pen strokes provided by the user inside the region of interest and the algorithm applied graph cuts technique iteratively in order to extract the region precisely. For the application of realistic hair manipulations, Chai et al. [13, 14] proposed to combine semiautomatic hair segmentation method with strand tracing technique in order to construct 3D hair models. Similar to the method in [12], it also needs several user strokes inside the hair regions during the hair segmentation steps of [13, 14].

**Figure 1:**
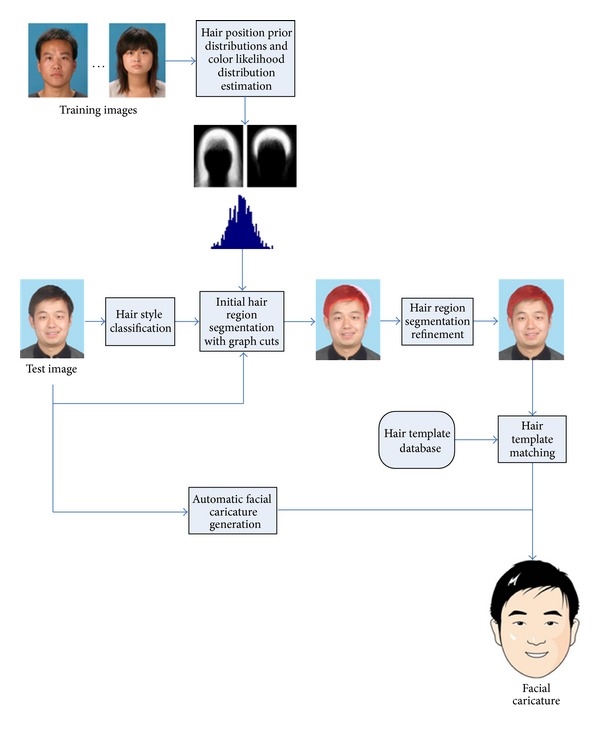
Workflow of our proposed algorithm.

In this paper, we propose an efficient automatic hair segmentation algorithm by combining graph cuts optimization and K-means clustering, since the application of our proposed method is automatic facial caricature synthesis. The head pose can be controlled and nearly frontal. As a result, our proposed method aims at dealing with nearly frontal faces which is similar to the most existing methods. Detected hair region can be matched against hair templates database in order to get the closest one and generate the final facial caricature. In our algorithm, the training process is direct and very efficient. Our proposed algorithm works as follows: firstly, hair regions are labeled manually in the training set. Hair style is classified into long hair or short hair groups. We estimate hair position prior distribution images with regard to the above-mentioned two groups respectively. We also estimate hair color likelihood distribution function with training images. Then we estimate hair style of the test image according to image thresholding technique. Energy function is constructed according to the hair position prior distribution image and hair color likelihood distribution function. Graph cuts algorithm is applied in order to optimize the energy function and the initial hair region is estimated. Finally, we estimate the precise hair region through K-means clustering and post-processing techniques with the initial hair region. Figure 1 shows the workflow of our proposed algorithm. Experimental results proved that the average processing time of our proposed algorithm on our constructed test database is about 280 ms which is quite fast. The detection accuracy is above 90%. We also integrated our algorithm into an automatic facial caricature synthesis system and further experiments showed that the resulting facial caricatures are vivid and satisfying.

## 2. Notations

In this paper, **I**(**x**) stands for the color value of the pixel **x** = [*u*,*v*]^*T*^ in the image. We use YCbCr color space instead of the traditional RGB color space throughout this paper unless stated otherwise. That is to say **I**(**x**) = [Y(**x**),Cb(**x**),Cr(**x**)]^*T*^. We define *f*(**x**) = *f*_**x**_ to be the label at pixel **x**. In this paper, *f*_**x**_ ∈ {0, 1} in which 1 stands for nonhair region and 0 stands for hair region. The aim of our proposed algorithm is to get the optimal labeling strategy *f*_**x**_ given an image which contains a human face. We define *P*(*f*_**x**_) to be the prior distribution of label position and *P*(**I**(**x**) | *f*_**x**_) is the color likelihood of the label.

## 3. Hair Segmentation and Its Application of Automatic Facial Caricature Synthesis

### 3.1. Hair Position Prior Distributions and Color Likelihood Distribution Estimation

**Figure 2:**
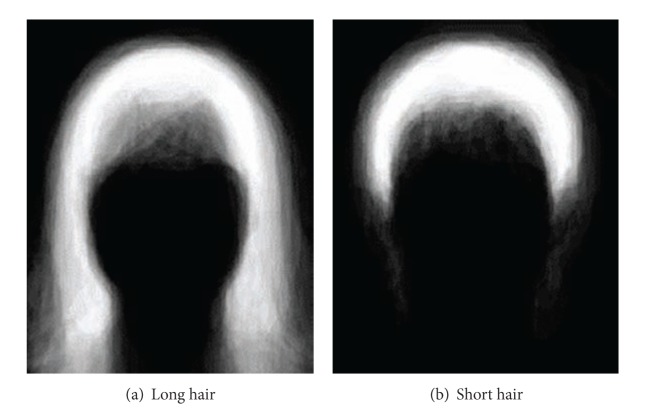
Prior distribution images for hair location. Images are normalized for viewing purpose. The brighter the pixel is the higher the probability is for this pixel belonging to hair region.

For the training image, we use face detection [15] algorithm to detect face region in the image. Then we expand the face region as described in [7] so that the detected region contains the hair region and normalize the image resolution into 200 × 250. For each normalized training image, we label hair region manually. In order to estimate *P*(*f*_**x**_) at every pixel **x**, we can set it to be the ratio between the number of images in which the pixel **x** belongs to hair region and the whole number of training images. Unfortunately, hair styles differ widely among different people. As a result, we calculate *P*_*l*_(*f*_**x**_) and *P*_*s*_(*f*_**x**_) separately corresponding to long hair and short hair styles. In this paper, we define that if the lower border of the hair is below the chin, the hair belongs to long hair style. Otherwise, it belongs to short hair style.  Figure 2 shows prior distribution images for hair location corresponding to long hair and short hair styles, respectively.

Hair color likelihood distribution *P*(**I**(**x**) | *f*_**x**_ = 0) is described with the YCbCr color space information which belongs to labeled hair region. We downsample each of Y, Cb and Cr channels into 64 levels. Then we can construct a three-dimensional color histogram in which each dimension has 64 bins. We normalize the color histogram in order to approximate the hair color likelihood distribution. Since hair color usually concentrates in a compact region in color space, we can store the nonzero bins in the color histogram in order to reduce the memory space consumption during implementation. Nonhair color likelihood distribution *P*(**I**(**x**) | *f*_**x**_ = 1) is approximated with uniform distribution.

### 3.2. Hair Style Classification

Since we construct *P*_*l*_(*f*_**x**_) and *P*_*s*_(*f*_**x**_) separately corresponding to long hair and short hair styles, we have to estimate the hair style for every test image.

We get the head region in the test image similarly as described in Section 3.1. Then the head image is normalized into 400 × 500. (In this paper, we segment hair region at this resolution.) Our proposed hair style classification algorithm is as follows.

**Step 1**

**Figure 3:**
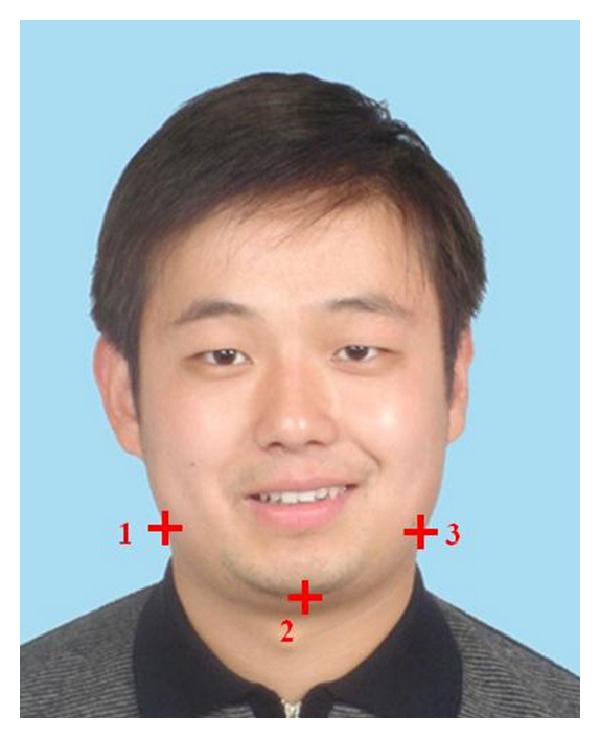
Three feature points detected by ASM algorithm. They are 2 intersection points between neck and face and the most bottom point of the chin is labeled by crosses with indices.

Detect 3 feature points **F** **P**_1_ = [*x*_1_,*y*_1_]^*T*^, **F** **P**_2_ = [*x*_2_,*y*_2_]^*T*^ and **F** **P**_3_ = [*x*_3_,*y*_3_]^*T*^ as shown in Figure 3 with ASM [16].

**Step 2**

We convert the color image into gray scale image and construct its corresponding histogram hist. We set the segmentation threshold *t* according to the following equation:

(1) $$\begin{array}{c} t=\underset{k}{\arg\;\max}\sum_{i=0}^{k}\text{hist}\left(i\right)<\alpha \cdot M\;\left(0\leq k\leq 90\right), \end{array}$$

where hist(*i*) is the number of pixels at brightness level *i* and *M* is the number of pixels in the face image. We set *α* = 0.2 according to experiments. We also set the threshold to be less than 90 in order to make sure that the threshold will not be too large when the hair region is small.

**Step 3**

Generate the face binary image **I**_*b*_ according to the threshold *t*. This means if the brightness value is below *t*, we set the value **I**_*b*_ to be 0. Otherwise, it is set to 1. We eliminate the noises in binary image using morphological filtering technique. In order to mitigate the problems caused by the dark collar, we apply the following equation:

(2) $$\begin{array}{c} I_{b}\left(x,y\right)=1\;\left(x_{1}⩽x⩽x_{3},\frac{\left(y_{1}+y_{3}\right)}{2}⩽y⩽H\right), \end{array}$$

where *H* is the height of the image. We calculate the number of pixels whose values are 0 in each scan line of the image and get the histogram hist_*v*_ in vertical direction.

**Step 4**

We classify the hair style according to the following equation:

(3) HS ={longhistv(i)>0,  ((y1+y3)2≤i≤(1−β)y2+βH)shortotherwise.

We have defined in Section 3.1 that if the lower border of the hair is below the chin, the hair belongs to long hair style. As a result, we detect whether there are continuous hair pixels from intersection point between neck and face to the position below the chin to determine the style of the hair. *β* controls the balance between detection accuracy and robustness. We set *β* = 0.2 according to experiments.

### 3.3. Initial Hair Region Segmentation with Graph Cuts

We firstly construct energy function and optimize it with graph cuts algorithm in order to get the initial segmentation results of the hair region. Energy function is defined as follows:

(4) $$\begin{array}{c} E\left(f_{x}\right)=\;E_{\text{data}}\left(f_{x}\right)+E_{\text{smooth}}\left(f_{x}\right). \end{array}$$

*E*_data_(*f*_**x**_) evaluates the likelihood between observations and labels:

(5) $$\begin{array}{c} E_{\text{data}}\left(f_{x}\right)=-\sum_{x}\left(\log P\left(I\left(x\right)\;|\;f_{x}\right)+\log P_{\text{sel}}\left(f_{x}\right)\right). \end{array}$$

*P*_sel_(*f*_**x**_) is defined as

(6) Psel(fx)={Pl(fx)HS=longPs(fx)HS=short.

*E*_smooth_(*f*_**x**_) evaluates the smoothness of the label results in the neighborhood:

(7) Esmooth(fx) =∑{xi,xj}∈NEδ(fxi≠fxj)exp⁡(−γ−1||Y(xi)−Y(xj)||2).

*δ*( ) is the Dirac delta function. NE is the set of all the 4-neighborhood systems in the image. *γ* is the normalization constant. In this paper we choose it to be the average contrast value of all the 3 × 3 blocks in the image.

We substitute (5) and (7) into (4) and get the final energy function. This energy function contains too many variables and could hardly be optimized through traditional gradient based optimization algorithm. In order to cope with this difficulty, we use graph cuts algorithm [17] to optimize this energy function. Graph cuts algorithm converts the energy function into a special graph data structure and converts the energy function optimization problem into the problem of getting the minimum cut of the graph. This problem could be solved efficiently according to the maximum flow method. Graph cuts algorithm has gained many successful applications in computer vision problems by now [18]. With graph cuts algorithm, we can get the approximate optimal solution of (4):

(8) $$\begin{array}{c} f_{x}^{*}\approx \underset{f_{x}}{\arg\;\min}\;E\left(f_{x}\right). \end{array}$$

**Figure 4:**
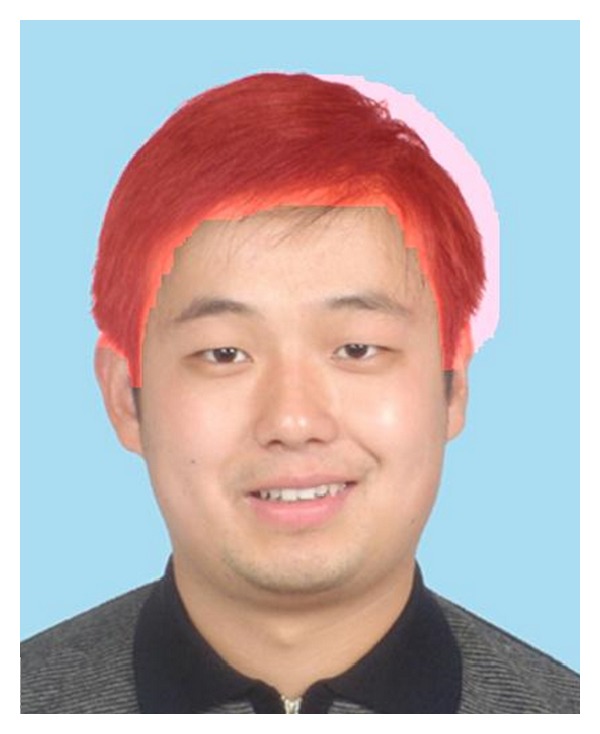
Initial hair region. Hair region is labeled in light red.

The computational cost of graph cuts algorithm is closely related to the resolution of the input image. For this reason, we downsample the resolution of the input image to 200 × 250 and estimate the approximate optimal hair segmentation result at this lower resolution. Then we upsample the hair region to the original resolution. This method greatly reduces the computational cost.  Figure 4 shows the estimated initial hair region of the subject in Figure 3 with the above described methods.

### 3.4. Hair Region Segmentation Refinement

From Figure 4 we can find that the initial hair region is contaminated with background and face region pixels. The reason for this is that the energy function contains smoothness term *E*_smooth_(*f*_**x**_) so that it tends to produce smooth hair region. Furthermore, we estimate the initial hair region at lower resolution. This will also cause problem to the accuracy of the detection results. In this paper, we propose to combine K-means clustering algorithm [20] and image postprocessing techniques to refine the initial hair region.

**Step 1**

**Figure 5:**
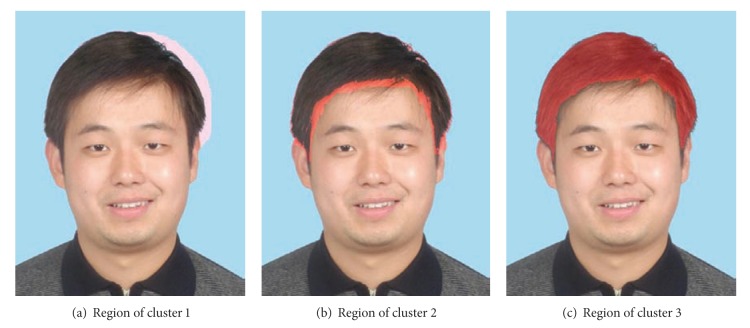
Three regions corresponding to three cluster centers generated by K-means algorithm. They are labeled in light red.

For all the pixels in the initial hair region, we cluster them into 3 groups with K-means clustering technique. Cluster centers of the 3 groups are **C**_1_ = [*R*_1_,*G*_1_,*B*_1_]^*T*^,  **C**_2_ = [*R*_2_,*G*_2_,*B*_2_]^*T*^,  and  **C**_3_ = [*R*_3_,*G*_3_,*B*_3_]^*T*^, respectively. Then we calculate their corresponding brightness values according to *Y*_*i*_ = 0.299*R*_*i*_ + 0.587*G*_*i*_ + 0.114*B*_*i*_  (*i* ∈ {1, 2, 3}).  Figure 5 shows the regions corresponding to 3 clusters, respectively. They are all labeled in light red. The reason for us to choose 3 clusters is as follows. Usually the initial hair region is contaminated with small regions in background and face region which are near the hair region. We can safely assume that the color information in these regions is relatively homogeneous. Thus we can divide the initial hair region into background, hair, and face subregions.

**Step 2**

Solve the following equation:

(9) $$\begin{array}{c} idx=\underset{i}{\arg\;\min}\;{\text{Y}}_{i}\;\left(i\in \left\{1,2,3\right\}\right). \end{array}$$

Hair region is usually with low brightness, so it is safe to assume that the pixels belonging to cluster *idx* are from hair region. Since it is possible that the initial hair region is good enough and does not contain background or face skin region, we have to determine whether the remaining pixels which belong to the rest 2 clusters are from hair region or not. If the following equation is satisfied, the pixels which belong to cluster **C**_*i*_ are from hair region:

(10) Yi<rYidx∧|Ri−Bi|<trb∧|Gi−Bi|<tgb(i∈{1,2,3}∧i≠idx).

Equation (10) permits certain amounts of inhomogeneity of brightness and color in the hair region. *r* controls the inhomogeneity of brightness in the hair region. It is determined by maximizing the hair detection accuracy in training images. In this paper, we set *r* = 3. *t*_*rb*_ and *t*_*gb*_ can be estimated through the following methods. We construct |*R* − *B*| and |*G* − *B*| histograms of the hair regions in training images. Similar to the 3*σ* rules in Gaussian distribution, we choose *t*_*rb*_ and *t*_*gb*_ in order to make sure that 95% of the pixels fall in these intervals. This improves the robustness in dealing with dyeing hair. In this paper, we choose *t*_*rb*_ = 40 and *t*_*gb*_ = 30.

**Step 3**

Hair region obtained from the above 2 steps may contain noises and holes. In this paper we label connected components in the hair region and calculate their corresponding area. We eliminate small regions whose areas are smaller than *t*_area_ and fill in the holes whose areas are smaller than *t*_area_. In this paper we choose *t*_area_ = 400. The final hair detection result of the subject in Figure 3 can be found in the first image of Figure 8.

### 3.5. Integration into Automatic Facial Caricature Synthesis System

**Figure 6:**
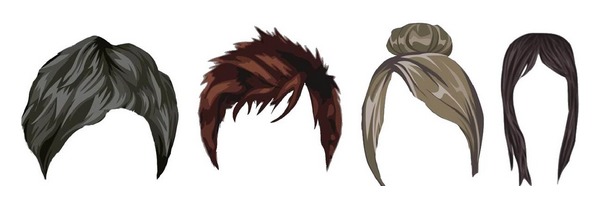
Cartoon hair template examples.

**Figure 7:**
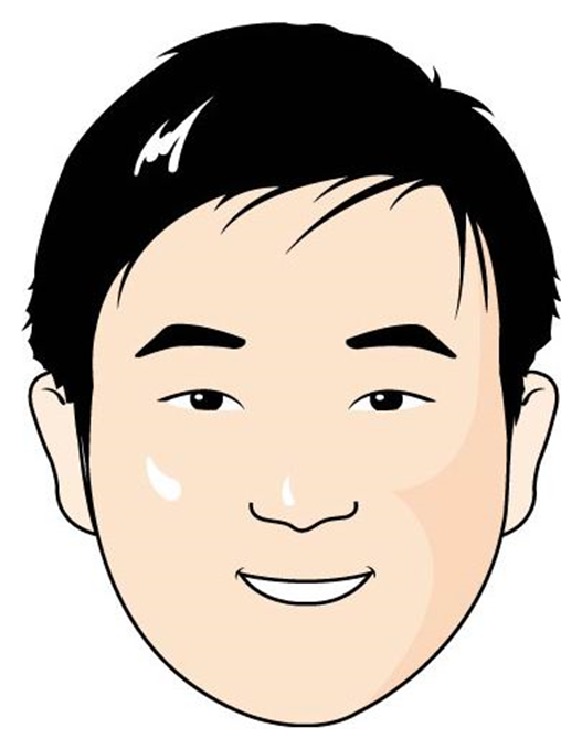
Automatic face caricature generation result of the person in Figure 3.

In order to integrate our proposed hair segmentation algorithm into automatic facial caricature synthesis system, we first construct a cartoon hair templates database with the help of professional cartoonists. Several cartoon hair templates in the database can be found in Figure 6. We construct 7-dimensional shape feature vectors for each hair template in the database by calculating its corresponding Hu moments [21]. For the test image, we segment hair region with the above-mentioned method. We also calculate its 7-dimensional shape feature vector. Then we find the best match in hair template database with minimum distance for the feature vectors. We scale the template image with the width of the detected hair region and shift it according to the gravity position of the detected hair region. For the rest part of the face (face region without hair), we generate the cartoon effect with the method described in [19]. In order to cope with the glasses in the human face, we detect the location and shape of glasses automatically with the method proposed in [22]. We also constructed a cartoon glasses templates database. The best glasses are again determined by comparing their corresponding 7-dimensional Hu moments. The scale and position of the glasses are determined according to the width of the face and the locations of the eyes in the face. This information could be easily extracted from the feature points detected in the face as described in [19].  Figure 7 shows the automatic face caricature generation result with our proposed method for the person in Figure 3.

## 4. Experimental Results

### 4.1. Hair Region Segmentation Results

#### 4.1.1. Image Database

**Figure 8:**
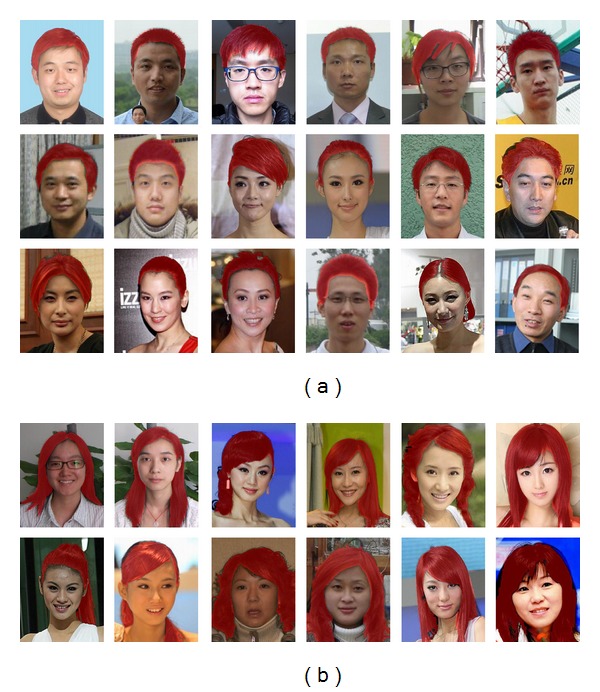
Examples of hair region detection results of our proposed method. The first 3 rows are short hair results and the remaining 2 rows are long hair results. Hair regions are labeled in light red.

We construct a database which contains 400 images. All the images in the database are downloaded from the Internet or captured by ourselves with consumer grade digital cameras. We separate them manually into 2 groups which correspond to long hair group and short hair group, respectively. We choose 40 images randomly from each group and construct the training set which contains 80 images and process them according to the method described in 3.1. The remaining 320 images are test images in which 137 of them are long hair ones and 183 of them are short hair ones. Some of the test images can be found in Figure 8. We also label the hair regions of these 320 images manually as benchmarks for quantitative analysis.

#### 4.1.2. Hair Segmentation Results

Figure 8 shows the hair segmentation results in some of the images from our constructed database with our proposed algorithm. The first 3 rows are results for short hair segmentation and the rest 2 rows are long hair segmentation results. Hair regions are labeled in light red. Similar to [7], we use FRR (false rejection rate) and FAR (false acceptance rate) for quantitative analysis.  Table 1 shows the results.

From Table 1 we can conclude that the average detection error is below 10% and the performances are acceptable. For the sake of comparisons, we implemented the method proposed in [7] by ourselves since the authors in [7] have not published their codes. The parameters used in [7] are set by minimizing the average FAR+FRR value in our test dataset. The results are shown in Table 2.

From Table 2 we can conclude that the average FRR value is quite similar and our algorithm is a little better. However, for the FAR value, our proposed method is much better which proves the effectiveness of our proposed algorithm.

#### 4.1.3. Hair Style Classification Results

Table 3 shows the hair style classification accuracies in long hair and short hair test sets, respectively.

In order to analyze the effect of hair classification on the final hair segmentation accuracy, we train a hair position prior image by mixing the short hair and long hair training images together. During the hair segmentation process, we also do not discriminate between the short and long hair styles.  Table 4 shows the hair segmentation accuracies under this configuration.

We also tested the hair segmentation accuracy with manually labeled hair style. The results are shown in Table 5.

From Tables 3, 4, and 5 we can find that if we do not consider the hair style, the hair segmentation accuracy degrades rapidly. As a result, hair style classification is necessary. If the hair style classification is perfect, the hair segmentation accuracy will be greatly improved. Considering Table 3 and comparing the results in Tables 1, 4, and 5 we can draw the conclusion that the accuracy of hair style classification algorithm proposed by us is quite high and greatly improves the hair segmentation results.

#### 4.1.4. Computational Cost Analysis

We implemented our algorithm with the help of OpenCV [23] under Microsoft Visual Studio 2005 and tested it on a laptop equipped with Intel Core2 Duo P7350 2.0 GHz CPU and 2 GB RAM. Details of every step in the algorithm are shown in Table 6.

The computational cost of our proposed algorithm is much lower than the method proposed in [7] and the performance of our proposed algorithm is better. This proves the effectiveness of our algorithm.

### 4.2. Automatic Facial Caricature Generation Results

**Figure 9:**
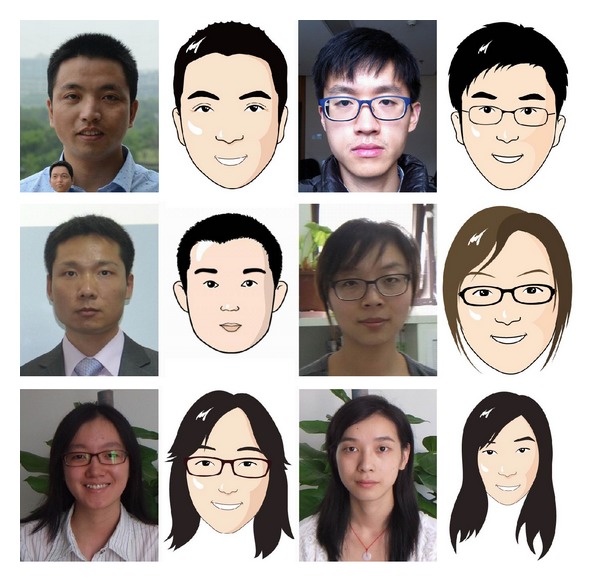
More examples of automatic facial caricature generation results with different styles [19] by our proposed system.

In order to verify the power of our proposed hair segmentation algorithm in automatic facial caricature generation system, we tested the 320 test images in our database with the automatic facial caricature generation system as described in Section 3.5. Several facial caricatures results could be found in Figures 7 and 9.

In order to analyze the effect of our proposed hair segmentation algorithm quantitatively, we conducted the following experiments. We recruited 5 people who have no ideas about our researches. They are 3 males and 2 females. All of them are between the ages from 25 to 35. For every test image in the database, we showed 4 images to each of them. The 4 images are comprised of 2 parts. The first image is the original test image and the rest 3 images are facial caricatures images. Among the 3 facial caricatures images, one of them was generated by our proposed system, one was generated by choosing the cartoon hair template randomly, and the final one was generated by choosing the cartoon hair template manually in order to best fit the input image. Five people graded the 3 face caricatures for each test image separately without discussing. The grades are from 1 point to 5 points. One point stands for the facial caricature which is very different from the original image and the person's identity could hardly be identified. Two points stand for the facial caricature which is different from the original image and there are only a few similarities between them. Three points stand for the fact that there are some differences between the facial caricature and the original image but the person's identity could be recognized. Four points stand for the facial caricature and the original image are similar and there are only a few differences. Five points stand for perfect facial caricature generation result and the facial caricature is vivid and very similar with the original image.  Table 7 shows the average grading results for each of the 5 people. From Table 7 we can conclude that the facial caricatures generated by our proposed method are very close to the results with manually selected hair template and much better than choosing the hair template randomly. This again justifies the effectiveness of our proposed algorithm.

## 5. Conclusion

This paper proposes a novel hair segmentation algorithm based on combining graph cuts optimization and K-means clustering. Our method could be incorporated into automatic facial caricature generation system without difficulties. Quantitative experimental results proved that the computational cost of our algorithm is relatively low and the hair segmentation results are robust and accurate. We can generate vivid and faithful facial caricatures with the help of our hair segmentation algorithm. In the future we plan to incorporate texture information [24] in order to improve the detection accuracy and robustness of the hair region.

## Figures and Tables

**Table 1:** Quantitative results of our proposed algorithm.

	Average FRR	Average FAR
Short hair group	9.91%	6.94%
Long hair group	9.43%	7.22%

**Table 2:** Quantitative results of the algorithm proposed in [7].

	Average FRR	Average FAR
Short hair group	10.04%	11.77%
Long hair group	9.88%	13.16%

**Table 3:** Hair style classification accuracy.

	Classification accuracy
Short hair	95.63%
Long hair	97.08%

**Table 4:** Quantitative results of the algorithm without considering the hair style.

	Average FRR	Average FAR
Short hair group	14.55%	20.10%
Long hair group	15.58%	15.21%

**Table 5:** Quantitative results of the algorithm with manually labeled hair style.

	Average FRR	Average FAR
Short hair group	9.57%	6.35%
Long hair group	9.21%	7.02%

**Table 6:** Computational cost analysis.

	Average computational time (ms)
Hair style classification	66
Initial hair region segmentation with graph cuts	50
Hair region segmentation refinement	167

Total	283

**Table 7:** Grading results by 5 people.

Person index	Average grades of the facial caricatures generated by our proposed system	Average grades of the facial caricatures generated by selecting the hair template manually	Average grades of the facial caricatures generated by selecting the hair template randomly
1	3.95	4.12	2.23
2	3.62	3.88	1.99
3	3.65	3.97	2.01
4	3.23	3.56	1.54
5	3.71	4.06	2.04
